# Pressurized Intra Peritoneal Aerosol Chemotherapy in patients suffering from peritoneal carcinomatosis of pancreatic adenocarcinoma

**DOI:** 10.1371/journal.pone.0186709

**Published:** 2017-10-19

**Authors:** Tanja Khosrawipour, Veria Khosrawipour, Urs Giger-Pabst

**Affiliations:** 1 Department of General Surgery & Therapy Center for Peritoneal carcinomatosis, St. Mary’s Hospital Herne, Ruhr University Bochum, Bochum, Germany; 2 Basic Research Laboratories of the Department of Surgery, St. Mary’s Hospital Herne, Ruhr-University Bochum, Bochum, Germany; China Medical University, TAIWAN

## Abstract

**Background:**

Patients suffering from peritoneal carcinomatosis of pancreatic adenocarcinoma were treated with Pressurized Intra Peritoneal Aerosol Chemotherapy (PIPAC), initial clinical findings are presented.

**Methods:**

Single institution, tertiary referral center certified for therapy of peritoneal disease. Prospective data collection of PIPAC therapy with doxorubicin 1.5 mg/m^2^ and cisplatin 7.5 mg/m^2^ of body surface delivered at intervals of six weeks. The outcome criteria were microscopic pathological response, survival and adverse events (v4.0 CTCAE).

**Results:**

A total of 20 patients (m/f = 3:1) with a mean age of 64.9 (range: 45.0 to 87.0) years underwent 41 PIPAC procedures without intraoperative complications. The mean number of PIPAC cycles was 2.1 (range: one to four). Ten patients with ≥ 2 PIPAC applications were eligible for histological analysis to assess carcinoma regression. Complete or high grade tumor regression was found in two (10%) and five (25%) patients, respectively. An overall median survival of 36.6 weeks after the first PIPAC application was observed. One patient died postoperatively due to small bowel obstruction. No CTCAE level 3 and 4 complications occurred.

**Conclusion:**

In about one third of patients, repeated PIPAC therapy did induce histological regression of systemic chemo-resistant PC of pancreatic adenocarcinoma. Prospective randomized trials are needed to further clarify any clinical impact of such observations.

## Introduction

The reported incidence of pancreatic adenocarcinoma (PAC) in western countries is increasing. With approximately 49,000 new cases diagnosed and 41,000 observed deaths in 2015 in the USA, this tumor entity has become the fourth and fifth leading cause of death in men and women, respectively [1]. While surgical resection remains the only possible curative option, local non-resectability and/or synchronous metastasis reduce eligibility of patients that undergo curative resection to less than 15% [2]. However, even after complete surgical resection, 80% of patients will suffer from postoperative tumor recurrence within the first two years following surgery [3–5]. A frequent site of recurrence is the peritoneum, as 40% to 50% of cases show peritoneal metastasis [6]. At present, palliative systemic chemotherapy is considered the treatment of choice for peritoneal metastasized patients. However, the prognosis remains poor, as the reported median overall survival is reported to be around eight months [7, 8].

It is evident that treatment options in such patients are still scarce and measures should be taken to develop more effective treatment strategies [9]. Pressurized Intra Peritoneal Aerosol Chemotherapy (PIPAC) is a novel therapeutic method to treat patients suffering from advanced PC. Ex-vivo, in animal as well as data obtained in human patients suggest a higher local drug biodisponibility, an optimized intra-abdominal drug distribution pattern and a better therapeutic index compared to antecedent data reported for liquid IPC [10–12]. Currently, PIPAC is not well documented in the medical literature to treat PC of PAC. We report our experience with PIPAC in such patients.

## Methods

### Patients and regulatory framework

Since April 2012, our institution (University Hospital of the Ruhr-University Bochum) is a certified referral center for the treatment of peritoneal carcinomatosis by the German Society for General and Visceral Surgery. Encouraging safety and feasibility data obtained from tumor entities of non-pancreatic origin prompted us to offer PIPAC therapy to patients suffering from PC of pancreatic origin. Prior to PIPAC therapy, each patient was evaluated at our multidisciplinary tumor board. The indication for PIPAC therapy was decided on a case-by-case individual basis. Only patients with histologically documented and progressive PC after or under evidence-based systemic chemotherapy or patients who did not qualify for systemic chemotherapy due to medical contraindications underwent PIPAC therapy. Although there were no strict exclusion criteria for PIPAC therapy, patients suffering from clinical signs of gastro-intestinal occlusion and/or a Karnofsky Index (KI) < 50% were excluded. We intended to deliver at least three PIPAC cycles separated by a six week time interval. The study was performed in line with the guidelines of the Declaration of Helsinki, and each patient was asked to give written informed consent for data collection as well as for publication of data in an anonymous manner. Data collection and analysis were performed with the approval of the local Institutional Review Board (Ethics Committee of the Ruhr University Bochum, Germany; Registry Number 15–5280)

### PIPAC procedure

The PIPAC procedure has previously been described in detail by our group [11, 13, 14]. The access to the abdominal cavity was usually obtained via a mini laparotomy just lateral to the left rectus muscle in the midclavicular line at the level of the umbilicus. Peritoneal biopsies from all four abdominal quadrants (if possible) were retrieved and consecutively prepared for histological analysis. A blunt 12 mm trocar was introduced and a constant capnoperitoneum of 12 mmHg established. A second trocar of 5 mm diameter was then placed under video-optic guidance. When present, ascites was completely removed and the amount documented. Then, a full inspection of the abdominal cavity was performed and the extent of PC (according to the Sugarbaker’s peritoneal carcinomatosis index) documented. Peritoneal biopsies from all four abdominal quadrants (if possible) as well as a local peritonectomy specimen of 3.0 x 3.0 cm in size were retrieved and consecutively prepared for histological analysis. For an optimum of intraperitoneal drug aerosol distribution, the PIPAC-Micropump (MIP^®^ Micropump/Capnopen (Reger Medizintechnik, Rottweil, Germany)) was inserted into the 12mm trocar with a maximum spraying distance between the MIP^®^ nozzle orifice and the small bowel [15, 16]. Doxorubicin at a dose of 1.5 mg/m^2^ body surface in a 50 ml NaCl 0.9% followed by Cisplatin at a dose of 7.5 mg/m^2^ in a 150 ml NaCl 0.9% were aerosolized and consecutively applied. The therapeutic aerosol was maintained at 12 mmHg for 30 min at 37 C. Finally, the aerosol was evacuated from the abdominal cavity using a Closed Aerosol Waste System, trocars were then retracted and laparoscopy ended. Previous reported safety standards for PIPAC therapy were followed [17].

### Data collection follow-up and statistical analysis

Data were collected by clinical study nurses according to a prospective data base including electronic archiving and video recording of the PIPAC procedures. Follow-up data was obtained by telephone calls (UGP) until 1^st^ July 2017 or until death occurred. Histological tumor response was assessed by an independent pathological reference center. To evaluate the histological tumor regression grade (TRG) associated with PIPAC therapy, the following criteria were applied: TRG I: < 10% to no tumor cells destroyed; TRG II a: 10% to 50% of tumor cells destroyed; TRG II b: 51% to 90% of tumor cells destroyed; TRG III: > 90% of tumor cells destroyed and TRG IV: no viable tumor cells with acellular pools of mucin [18]. In this study, whenever different scores were found in different tissue samples of the same patient, the lowest TRG value observed was reported. Adverse events were graded according to the Common Terminology Criteria for Adverse Events (v4.0 CTCAE) [19]. Data analysis was conducted retrospectively. Overall survival was modelled in a Kaplan-Meier curve with IMB^®^ SPSS^®^ Statistics 24.

## Results

Between June 2015 and June 2017, a total of 20 patients (m:f = 3:1) with a mean age of 64.9 years (range: 45 to 87) and a median Karnofsky Index of 80% (IQR: 70% to 90%) underwent a total of 41 consecutive PIPAC applications. Eight patients had undergone previous curative pancreatic resection but developed histologically confirmed metachronous PC. In another twelve patients, diagnostic laparoscopy/laparotomy confirmed synchronous PC. All patients had progressive PC under or after a minimum of one line of systemic chemotherapy or systemic treatment had to be stopped due to severe systemic side effects. The median time interval between the diagnosis of pancreatic adenocarcinoma and the first PIPAC application was 331 (IQR: 170.5 to 472.5) days. Patient demographic data as well as details on previous surgical procedures and systemic chemotherapy are summarized in Table 1.

**Table 1:** Patient demographic and details on previous surgical interventions and systemic chemotherapy treatment before PIPAC.

Pat.N°	Age (yrs)	Sex	Date of diagnosis of PAC (mm/yy)	Type and date of previous surgery (mm/yy)	Details about previous systemic chemotherapy treatments	ECOG prior to PIPAC N° 1	Days between diagnosis of PAC & 1. PIPAC	Comments
**1**	68	f	08/14	Pancreatic tail resection 12/14	7 x Gemcitabine and nab-Paclitaxel	0	90	Progress under systemic therapy; PIPAC mono
**2**	74	m	01/14	DL 01/14	12 x Gemcitabine and nab-Paclitaxel	1	465	Progress after systemic therapy; PIPAC mono
**3**	59	f	05/14	WR 05/15	12 x FOLFIRINOX, 5 x Gemcitabine Gemcitabine & Erlotinib	0	510	Progress under second-line systemic therapy; PIPAC mono
**4**	54	m	12/15	DL 12/15	8 x Gemcitabine	1	85	Progress under systemic therapy; PIPAC mono
**5**	65	m	03/15	Pancreatic tail resection & splenectomy 04/15	5 x FOLFIRINOX Gemcitabine & nab-Paclitaxel	0	360	Gemcitabine & nab-Paclitaxel and PIPAC
**6**	61	m	03/15	DL 03/15	8 x FOLFIRINOX	0	480	Progress after systemic therapy; PIPAC mono
**7**	63	m	10/14	DL 10/14	7 x FOLFIRINOX	0	150	Progress under systemic therapy; PIPAC mono
**8**	45	f	12/14	WR 01/15	4 x Gemcitabine	0	270	Stop of systemic therapy due to neutropenia; PIPAC mono
**9**	55	f	03/14	WR 03/15	1 x FOLFIRINOX 6 x Gemcitabine	1	480	Stop FOLFIRINOX due to side effects; progress under Gemcitabine; PIPAC mono
**10**	69	m	02/15	DL 02/15	3 x FOLFIRINOX	1	210	Stop Folfirinox due to side effects; Patient refused any systemic therapy; PIPAC mono
**11**	62	m	04/15	Thoracoscopy and Wedge resection 04/15 DL 07/16	2 x Gemcitabine & nab-Paclitaxel 6 x FOLFIRINOX	1	455	Synchronous lung metastasis; PIPAC and FOLFIRINOX (80%) combined
**12**	62	m	02/13	WR 02/13	Gemcitabine mono12 x FOLFIRINOX 4 x Gemcitabine & nab-Paclitaxel	1	1290	PIPAC mono
**13**	87	m	11/15	DL 11/15	Gemcitabine mono	1	17	Progressive disease under systemic therapy; kidney insufficiency; PIPAC mono
**14**	52	f	6/16	DL 10/16	6 x Gemcitabine & nab-Paclitaxel 3 x FOLFIRINOX	1	191	Folfirinox and PIPAC
**15**	71	m	05/16	DL 05/16	22 x Gemcitabine & nab-Paclitaxel	1	350	Stop systemic chemotherapy due to progressive disease; PIPAC mono
**16**	72	m	01/17	DL 01/17	5 x Gemcitabine & nab-Paclitaxel 2 x FLOX	1	52	Duodenal stent placement prior to PIPAC;; pulmonary metastasis under Gemcitabine & nab-Paclitaxel; Stop FLOX due to severe side effects PIPAC mono
**17**	61	m	03/13	DL 03/13	10 x FOLFIRINOX, 10 x Gemcitabine & nab-Paclitaxel, 5 x Cisplatin & Gemcitabine	2	1439	Progressive disease; kidney insufficiency; PIPAC mono
**18**	75	m	06/16	Pancreatic tail resection & splenectomy 06/16	1 x FOLFIRINOX 6 x Gemcitabine & nab-Paclitaxel	0	312	Gemcitabine & nab Paclitaxel & PIPAC
**19**	70	m	04/16	Explorative laparotomy 04/16	22 x Gemcitabine & nab-Paclitaxel; Gemcitabine mono	1	423	Synchronous PC and liver metastasis; PIPAC and Gemcitabine mono
**20**	72	m	06/14	WR 06/14	6 x Gemcitabine mono	1	299	PC and synchronous lung metastasis; PIPAC and Gemcitabine mono

yrs = years; m = male; f = female; PAC = pancreatic adenocarcinoma; WR = Whipple resection; DL = diagnostic laparoscopy; KI = Karnofsky Index

With an initial mean PCI score of 26.6 (range: 1 to 39) and malignant ascites of 1403 ml (range: 0 to 7000), all patients received a minimum of one PIPAC application. In three patients, safe access to the abdominal cavity for a second PIPAC application failed due to severe adhesions—representing a secondary non-access rate of 3/41 (7.3%) procedures. The mean number of PIPAC applications was 2.1 (range: one to four) whereas four patients received four applications, three patients three, three patients two and ten patients only one. The mean procedure time was 93.0 (range: 75 to 125) minutes with no observed intraoperative complications. Mild postoperative abdominal discomfort and pain (CTCAE grade 1) was recorded for 14/41 (34%) procedures. Profuse nausea and vomiting (CTCAE grade 2) was observed in one case (2.4%). No CTCAE grade 3 and 4 complications occurred. Although not in direct correlation with the PIPAC procedure itself, one patient died eleven days after the first PIPAC application (CTCAE grade 5) due to small bowel obstruction which represents a procedure related mortality rate (1/41) of 2.4%.

Out of twenty patients, ten patients received ≥ 2 PIPAC cycles and were therefore eligible for histological tumor regression grade (TRG) analysis. In two patients (10%), all biopsies as well as in the local parietal peritonectomy specimen, complete tumor regression (TRG grade 4) was observed. Furthermore, high grade tumor regression (TRG 3) was documented in five patients (25%). No histological response to PIPAC therapy was observed in three (15%) patients (TRG grade 1). Taken together, any objective histological tumor regression was observed in 7/20 (35%) patients.

**Fig 1:**
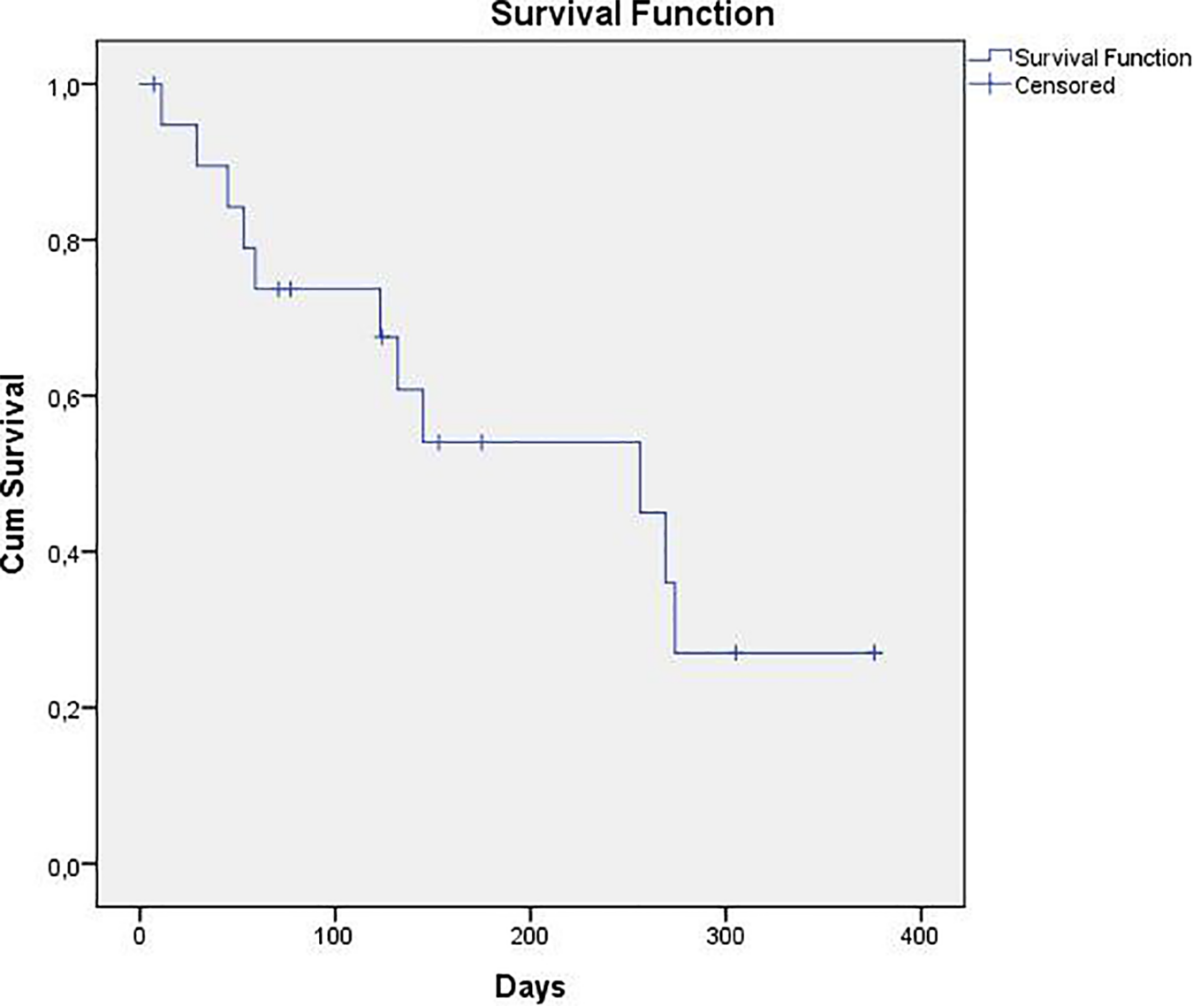
Overall survival curve Kaplan-Meier after first PIPAC application.

The reasons for premature interruption of PIPAC therapy (minimum of three cycles) were rapid physical deterioration with clinical and/or radiological signs of subileus/ileus, severe tumor cachexia syndrome or exitus letalis in eight patients (40%) as well as secondary non-access in three other patients (15%). On an intention-to-treat analysis, one third of patients could be managed with at least three PIPAC therapies, two patients are awaiting for their third PIPAC cycle. The median survival after the first PIPAC cycle was 36.6 weeks (95% CI: 36.6–51.1 weeks). After a median follow up of 43.6 weeks (95% CI: 8.5–78.6 weeks), eleven patients have died. Details about PIPAC therapy, perioperative complications, and histological regression are summarized in Table 2. Survival data after the first PIPAC cycle are given in Fig 1.

**Table 2:** Perioperative details about PIPAC therapy.

Pat. N°	PIPAC N° 1				PIPAC N° 2				PIPAC N° 3				PIPAC N° 4				Comments:
	PCI Score	Ascites (ml)	CTCAE 1–5	TRG 1–4	PCI Score	Ascites (ml)	CTCAE 1–5	TRG 1–4	PCI Score	Ascites (ml)	CTCAE 1–5	TRG 1–4	PCI Score	Ascites (ml)	CTCAE 1–5	TRG 1–4	
**1**	2	0	1	1	16	50	0	2	23	800	1	3	-	-	-	-	Exitus letalis 08/15 before PIPAC N° 4
**2**	20	3000	5	1	-	-	-	-	-	-	-	-	-	-	-	-	Exitus letalis 06/15 eleven days after PIPAC N° 1 due to postoperative ileus
**3**	7	0	1	1	9	0	0	2	15	0	0	4	***	0	1	2	Exitus letalis 08/16
**4**	5	3000	0	1	-	-	-	-	-	-	-	-	-	-	-	-	Rapid clinical deterioration after PIPAC N° 1; Exitus letalis 02/16
**5**	10	30	1	1	-	-	-	-	-	-	-	-	-	-	-	-	Secondary non-access after PIPAC N° 1 due to adhesions; Exitus letalis 11/16
**6**	1	50	0	1	6	30	1	3	5	0	0	3	7	50	1	4	Alive 06/17 with no chemotherapy
**7**	20	4500	0	1	18	800	0	1	-	-	-	-	-	-	-	-	Exitus letalis 06/16; rapid clinical deterioration after PIPAC N° 2
**8**	2	0	0	1	2	0	1	3	17	30	0	3	-	-	-	-	Clinical deterioration after PIPAC N° 3; Exitus letalis 03/16
**9**	33	200	2	1	-	-	-	-	-	-	-	-	-	-	-	-	Secondary non-access due to adhesions; Exitus letalis 09/15
**10**	31	3500	1	1	25	5600	1	3	-	-	-	-	-	-	-	-	Exitus letalis 02/16; Stop PIPAC due to clinical deterioration
**11**	1	50	0	1	6	30	1	3	5	0	0	3	2	50	0	3	Alive 06/17 but clinical deterioration
**12**	24	50	0	1	21	50	1	1	24	0	1	1	24	0	0	1	Alive 06/17; no treatment;
**13**	27	7000	0	1	-	-	-	-	-	-	-	-	-	-	-	-	Stop PIPAC due to clinical deterioration; Exitus letalis 01/17
**14**	5	100	0	1	-	-	-	-	-	-	-	-	-	-	-	-	Exitus letalis 05/17 due to sepsis
**15**	16	80	0	1	-	-	-	-	-	-	-	-	-	-	-	-	Alive 06/17; stop PIPAC due to subileus
**16**	29	1500	0	1	17	0	0	2	17	0	0	3	-	-	-	-	Alive 06/17
**17**	39	3500	0	1	-	-	-	-	-	-	-	-	-	-	-	-	stop PIPAC due to rapid disease progress; Exitus letalis 04/17
**18**	23	1000	0	1	-	-	-	-	-	-	-	-	-	-	-	-	Secondary-non access due to adhesions; alive 06/17
**19**	3	500	1	1	7	700	0	1	1	-	-	-	-	-	-	-	Third PIPAC scheduled; alive 06/17
**20**	5	0	0	1	-	-	-	-	-	-	-	-	-	-	-	-	Exitus letalis 09/16

TRG 1–4 = tumor regression grade (1 = 10% to no tumor cells destroyed; 2 = 10% to 50% of tumor cells destroyed; 3 = 90% of tumor cells; 4 = no viable tumor cells and acellular pool of mucin); PCI = Sugarbaker’s peritoneal carcinomatosis index; N/A = not applicable; CTCEA = Common Terminology Criteria of Adverse Events (v4.0); Pat. N° = patient number

*** = PCI not determinable due to diffuse sclerosis of peritoneum

## Discussion

Systemic chemotherapy has poor efficacy and often severe toxic side effects, questioning its clinical use [8, 20]. Inadequate drug delivery to solid tumors is a major cause of treatment failure. Following systemic administration, the drug delivery to the cancer cells in solid tumors involves transport within a vessel, transport across the vascular wall into surrounding tissue and transport through the intestinal space within the tumor. These processes are determined by the physiochemical properties of the drugs and the biological properties of the tumor. Among the peritoneal tumors, drug delivery to pancreatic cancer is problematic, owing to the high stromal fraction (> 80%) resulting in a high interstitial pressure and in the sparse vascular that is only partially functional and physically separated from cancer cells [21].

Based on such data, intraperitoneal chemotherapy (IPC) represents a logical alternative route for delivering high drug concentrations to tumors located in the peritoneal cavity. A combination of liquid IPC and intravenous chemotherapy for the treatment of systemic gemcitabine refractory PC of PAC has already been tested. A median overall survival of 4.8 months and a high number of CTCEA grade 3 and 4 adverse events such as neutropenia (34.0%), anemia (31.0%), and catheter-related infections (9.0%) have been reported [22]. Even with such a combined and more aggressive therapy, a high number of severe toxic side effects are observed and the overall survival remains poor.

Although the pharmacokinetics of PIPAC and liquid IPC have never been directly compared and there is a lack of evidence about the optimum drug dose, duration between therapy intervals, pressure of the capnoperitoneum, and exposure time for PIPAC therapy, PIPAC has rapidly gained wide acceptance in the daily management of patients suffering from PC of gastro-intestinal and gynecological origin.

Published data on safety and feasibility from our PIPAC program, as well as data published from other independent groups mainly based on one phase II study for recurrent PC of ovarian cancer and several retrospective case series of peritoneal metastatic gastric or colo-rectal origin, report a high feasibility and safety profile of PIPAC therapy. In this current series, aside from one patient suffering from profuse nausea and vomiting requiring prolonged intravenous fluid resuscitation and anti-emetic therapy (CTCAE grade 2), adverse events were self-limiting abdominal discomfort and perioperative nausea (CTCAE grade 1). No systemic side effects such as deterioration of kidney and/or liver function or myelosuppression were observed. Although one patient died after the first PIPAC cycle from small bowel obstruction due to progressive disease, the safety and feasibility data of PIPAC therapy observed in this current study are in line with previous reports on PIPAC therapy for PC of other origin than pancreatic cancer [23].

A key element of PIPAC is repetitive diagnostic staging laparoscopy which allows quantification of PC by visual inspection (PCI score) and objective histological therapy response by multiple biopsies. However, staging laparoscopy is known to underestimate the extent of PC, especially in patients with adhesions due to previous surgery such as 40% in this series. In our experience, another limiting factor for correct PCI documentation is the formation of varying but also extensive degrees of diffuse peritoneal sclerosis after repetitive PIPAC applications. This phenomenon has already been described after liquid intraperitoneal instillation for a variety of different cytostatic drugs [24]. Therefore, the PCI score is an invalid tool to monitor any therapy response of repetitive PIPAC applications.

However, patients who had a minimum of two PIPAC cycles were accessible for histological analysis of the tumor response. An objective tumor regression was observed in 35% of patients. Nevertheless, previous studies with patients suffering from end-stage PC of gastric or colo-rectal cancer reported a higher number of objective histological regressions in 50% to 70% of cases [13, 14].

In a very recently published small pilot study by Graversen et al., histological regression of first-line systemic chemotherapy resistant PC of pancreatic adenocarcinoma after PIPAC application was observed in 75% of patients [25]. However, these data must be interpreted with caution. First, it is a small pilot study dealing with only five patients. Second, their systemic first-line chemotherapy is not the standard in a western patient population. Finally, histological tumor regression was assessed with a new and until now never used or clinically validated peritoneal carcinomatosis regressions grading score [26]. Nevertheless, the data of Graversen et al. are important since these data are the first of its kind which report about an activity of PIPAC on systemic chemo-resistant PC of pancreatic origin and furthermore support our findings.

We are aware, that our current data needs to be interpreted with caution. First, this study is retrospective with only a small number of patients included. Second, patients with a rapid disease progression and deterioration of their general condition were not referred to our clinic for possible PIPAC therapy. Therefore, a certain selection bias to physically fitter patients treated with PIPAC must be assumed.

Currently it remains unclear whether histological regression due to PIPAC does have any clinical impact. Nevertheless, such data observed in end-stage PC patients are encouraging. In a phase II study combining intravenous and intraperitoneal liquid chemotherapy in gemcitabine refractory PC of PAC, Takahara et al. reported about a median overall survival of 4.8 months but with a high number of severe toxic side effects [22]. This contrasts with our current study with a median survival of nine months observed after the first PIPAC cycle and with almost no severe complications. Existing data on a combination therapy with systemic chemotherapy and PIPAC are furthermore promising. PIPAC was found not to further enhance severe toxic side effects already observed for systemic chemotherapy [13, 27]. Such a combination approach could be a further step towards improving the clinical outcome without increasing the risk of more severe side effects.

## Conclusion

PIPAC therapy in patients suffering from end-stage PC or pancreatic adenocarcinoma is feasible and safe as reported in earlier studies on ovarian and other gastro-intestinal tumor entities. However, the number of patients with an observed objective histological tumor regression in this study is inferior compared to that reported for PC of gastric or colo-rectal cancer treated with PIPAC. Further studies are needed to clarify if PIPAC has a possible positive impact on the clinical outcome of such patients.
